# Impact of COVID-19 on Dental Practices in El Salvador and Mexico: A Comprehensive Survey Analysis

**DOI:** 10.7759/cureus.46524

**Published:** 2023-10-05

**Authors:** Nuria Patiño-Marín, Wendy Yesenia Escobar de González, Katleen Argentina Aguirre de Rodríguez, Miguel Angel Casillas Santana, Carlo Eduardo Medina-Solís, Guillermo Alfonso Aguirre Escobar, Gabriel Alejandro Martínez-Castañón, Marco Salas

**Affiliations:** 1 Clinical Research, University of San Luis Potosí, San Luis Potosi, MEX; 2 Dentistry, Faculty of Dentistry, Universidad de El Salvador, San Salvador, SLV; 3 Orthodontics, Faculty of Stomatology, Meritorious Autonomous University of Puebla, Puebla, MEX; 4 Dentistry, Institute of Health’s Sciences, Autonomous University of the State of Hidalgo, Hidalgo, MEX; 5 Dentistry, Faculty of Dentistry, University of El Salvador, San Salvador, SLV; 6 Dentistry, Program of Doctorate in Dental Sciences, University of San Luis Potosi, San Luis Potosi, MEX; 7 Dentistry, Universidad Autonoma de San Luis Potosi, San Luis Potosi, MEX

**Keywords:** prevention, covid-19, protective barriers, prevention measures, dental care service

## Abstract

Background and objectives: This study aimed to identify the relationship between prevention measures and protective barriers in dental practice in El Salvador and Mexico during the COVID-19 pandemic in 2020 and 2021.

Materials and methods: A longitudinal study was conducted from June 2020 to December 2021, involving 1,719 dentists divided into four groups based on location and year. A 20-question survey in Spanish was utilized and validated with a Cronbach's alpha value of 0.84.

Results: The use of phone triage (OR = 1.3), thermometers (OR = 1.4), physical distancing (OR = 1.7), and face shields (OR = 2.6) was significantly associated with dental practice in both countries during the pandemic.

Conclusions: During 2020 and 2021, dental care in El Salvador and Mexico was significantly linked to COVID-19 preventive measures. Phone triage, thermometers, distancing, and face shields positively correlated with dental services. National health agencies should promote the use of minimum preventive measures in dental care, preparing for potential reinfections or new pandemics from emerging virus variants.

## Introduction

In January 2020, the Chinese Illness Prevention and Control Center officially announced a coronavirus as the pathogen causing COVID-19 [1]. Since then, COVID-19 has emerged as a significant public health issue, altering lifestyles worldwide [2]. As of September 2022, 609 million cases had been identified, and 6.5 million deaths were reported globally [3]. Various health professions have been deemed high-risk due to virus transmission. Consequently, modifications in health services and the implementation of preventive measures and protection barriers in clinical practice became imperative [4].

Dental interventions, including direct or indirect contact with mouth tissues, instrument handling, and aerosol generation, pose risks for COVID-19 transmission among dentists and patients [4,5]. As a preventive strategy, the World Health Organization (known as OMS in Spanish), Guidance for Dental Settings, and the Cochrane community initially recommended restricting routine oral health services to emergencies in many world regions [6-8]. Subsequently, governments worldwide established prevention protocols related to patient services as part of their pandemic initiatives [9].

In 2020, standard infection control measures in routine clinical practices seemed inadequate to halt the spread of COVID-19. Health professionals had to adapt their clinical practices to serve patients during the pandemic [10].

Some of these preventive measures included reducing the number of dental service locations, using phone triage, infrared thermometers, handwashing, maintaining physical distancing (at least one meter between patients), employing techniques to prevent aerosol generation, and surface disinfection [11].

Additionally, protective barriers, such as gloves, fluid-resistant face masks, safety goggles or face shields, disposable surgical caps, gowns, and personal protective equipment, were recommended to prevent contagion [12]. These changes have profoundly affected health professionals and patients worldwide.

The moment is suitable for 1) identifying information related to prevention measures, 2) analyzing and reevaluating preventive measures and protection barriers used in clinical practices, and 3) proposing and establishing prevention-based protocols to enhance dental services. Thus, this study's objective was to identify the association between dental care service and prevention measures and protective barriers used in clinical practice in El Salvador and Mexico during COVID-19 in 2020 and 2021.

## Materials and methods

A longitudinal study was conducted from June 2020 to December 2021 in El Salvador and Mexico. Participants voluntarily decided to partake in the survey. Participant anonymity was ensured to maintain the privacy and confidentiality of all collected information. The study received approval from the Research Ethics Committee of the Autonomous University of San Luis Potosi, San Luis Potosi, Mexico, and the Research Ethics Committee of the University of El Salvador, San Salvador, El Salvador.

Study population

The population comprised dentists working in clinics, hospitals, or health centers in El Salvador and Mexico.

Sample selection

The study instrument (questionnaire) was disseminated through various Facebook groups across all Mexican states (32 states) and all Salvadoran departments (14 departments). At least one Facebook group of dentists from each Mexican state and each Salvadoran department participated in the study. Consecutive non-probabilistic sampling was employed. The states and departments from Mexico and El Salvador were categorized into three regions: Central, North, and South. Ultimately, the questionnaire was accessible via a link to the Google Forms platform.

Sample size

Sample Size Calculation in Mexico

The required minimum sample size was 347, with an estimated total of approximately 70,000 dentists, a 50% probability, a 3% margin of error, and a 99% confidence level.

Sample Size Calculation in El Salvador

The required minimum sample size was 293, with an estimated total of approximately 1,800 dentists, a 50% probability, a 3% margin of error, and a 99% confidence level. The samples were determined using the sample size formula for finite populations.

Study instrument (questionnaire design)

After reviewing national and international publications, 16 questions in Spanish were formulated (Appendices). The survey assessed variables such as sociodemographic characteristics, dental care service, prevention measures, and personal protection barriers. Responses were dichotomous (Yes or No). The questionnaire underwent pre-testing for validity and reliability with 30 dentists, achieving a satisfactory Cronbach’s alpha value of 0.84 (p < 0.05).

Two periods of time (longitudinal study)

The questionnaire was distributed during two periods in El Salvador and Mexico: first, between June and July 2020, and second, between October and December 2021.

Statistical analysis

In the univariate analysis, categorical variables were presented as frequencies and percentages, while continuous variables were expressed as means and standard deviations. The bivariate analysis utilized the Chi-square test to determine differences between groups. A binary logistic regression analysis was constructed with dental care service as the dependent variable (1 for presence and 0 for absence). Independent variables included infrared thermometer, phone triage, handwashing, physical distancing (at least one meter between patients), face shield, and disposable gown.

A variance inflation factor (VIF) analysis test was conducted to detect and mitigate multicollinearity among the independent variables. The specification error test verified the assumption that the response variable's logit is a linear combination of the independent variables. After establishing the main effects, interactions were tested, but none were significant. The model's overall fit was assessed using the goodness of fit test. The association between dependent and independent variables is presented as odds ratios (OR) with 95% confidence intervals (CI). P-values < 0.05 were deemed statistically significant. Data was analyzed using JMP ver. 15 (SAS Institute, Cary, NC) statistical software [13].

## Results

A total of 1,719 dentists participated in the study: 964 from El Salvador and 755 from Mexico. These health professionals were divided into four groups:

1. El Salvador 2020: 528 dentists of both genders (69%, n= 365 women) aged between 25 and 75 years (44 ± 10) responded to the survey in 2020.

2. El Salvador 2021: 436 professionals of both genders (67%, n= 292 women) aged between 25 and 74 years (44 ± 11) responded in 2021.

3. Mexico 2020: 358 dentists of both genders (59%, n= 210 women) aged between 23 and 76 years (37 ± 10) responded in 2020.

4. Mexico 2021: 397 professionals of both genders (67%, n= 268 women) aged between 23 and 69 years (35 ± 9) responded in 2021.

The study encompassed all states and departments from Mexico and El Salvador, which were categorized into three regions: central, north, and south. In the El Salvador 2020 group, 75% (n= 392) of dentists hailed from the central region, 11% (n= 59) from the north, and 14% from the south. In the El Salvador 2021 group, the distribution was 73% (n= 318) from the central region, 8% (n= 34) from the north, and 19% (n=84) from the south.

In Mexico, the 2020 group had 66% (n= 237) of dentists from the central region, 27% (n= 34) from the north, and 7% (n=84) from the south. The 2021 group had 48% (n= 191) from the central region, 36% (n= 143) from the north, and 16% (n= 63) from the south.

Regarding dental care service, Table 1 displays the dental care service during COVID-19 in El Salvador and Mexico for 2020 and 2021. In El Salvador, 30% (2020) and 40% (2021) of dentists halted their clinical practice. In Mexico, the percentages were lower, with 5% (2020) and 3% (2021) of dentists ceasing their clinical practice. Initially and throughout the COVID-19 contingency, dental services in El Salvador shifted, primarily catering to emergency cases (2020: 37%, 2021: 49%, p= 0.0001). In Mexico, 2020 saw a surge in emergency cases (78%, n= 277). However, by 2021, most dental services incorporated preventive measures and protection barriers (86%, n= 339). In both countries, the percentages of dental services provided by health professionals were high at the onset and during the contingency (El Salvador: 65%, Mexico: 96%).

**Table 1:** Dental care service during COVID-19 in El Salvador and Mexico in 2020 and 2021. Statistical test: Chi-square

Groups	El Salvador 2020 N (%)	El Salvador 2021 N (%)	Total	P-value	Mexico 2020 N (%)	Mexico 2021 N (%)	Total	P-value
Suspension of dental care	159 (30)	175 (40)	334	0.0001	18 (5)	13 (3)	31	0.0001
Emergency only	195 (37)	212 (49)	407	0.0001	277 (78)	45 (11)	322	0.0001
Dental care with preventive measures and protection barriers	174 (33)	49 (11)	223	0.0001	63 (17)	339 (86)	402	0.0001
Total	528 (100)	436 (100)	964		358 (100)	397 (100)	755	

The preventive measures taken in clinical practice for COVID-19 in El Salvador and Mexico during 2020 and 2021 can be observed in Table 2. Measures such as phone triage, use of thermometers, hand washing, physical distancing (maintaining at least one meter between patients), and techniques to prevent aerosol generation were commonly employed. In El Salvador, we observed high utilization rates for thermometers (2020= 93%, 2021= 84%), hand washing (2020= 72%, 2021= 97%), and physical distancing (2020= 69%, 2021= 90%). In Mexico, notable percentages were observed in the use of phone triage (2020= 88%), infrared thermometers (2021= 84%), hand washing (2020= 96%, 2021= 99%), and physical distancing (2021= 96%).

**Table 2:** Prevention measures in clinical practice during COVID-19 in El Salvador and Mexico in 2020 and 2021. Statistical test: Chi-square. Physical distancing: space of at least one meter between patients.

Groups	El Salvador 2020 (n=369) N (%)	El Salvador 2021 (n=261) N (%)	Total	P-value	Mexico 2020 (n=340) N (%)	Mexico 2021 (n=384) N(%)	Total	P-value
Phone triage								
Yes	169 (46)	98 (38)	267	0.0385	299 (88)	203 (53)	502	0.0001
No	200 (54)	163 (62)	363		41 (12)	181 (47)	222	
Infrared thermometer								
Yes	343 (93)	219 (84)	562	0.0004	170 (50)	322 (84)	492	0.0001
No	26 (7)	42 (16)	68		170 (50)	62 (16)	232	
Handwashing								
Yes	266 (72)	254 (97)	520	0.0001	326 (96)	381 (99)	707	0.0001
No	103 (28)	7 (3)	110		14 (4)	3 (1)	17	
Physical distancing								
Yes	254 (69)	236 (90)	490	0.0001	212 (62)	370 (96)	582	0.0001
No	115 (31)	25 (10)	140		128 (38)	14 (4)	142	
Use of techniques that prevent the generation of aerosols								
Yes	80 (22)	179 (69)	259	0.0001	64 (19)	236 (62)	300	0.0001
No	289 (78)	82 (31)	371		276 (81)	148 (38)	424	

Table 3 displays the use of personal protective barriers in clinical practice during COVID-19 in both countries for the years 2020 and 2021. In El Salvador, high utilization rates were noted for N95 face masks (2020= 95%, 2021= 97%), gloves (2020= 100%, 2021= 98%), safety goggles (2020= 100%, 2021= 96%), surgical caps (2020= 100%, 2021= 97%), and face shields (2020= 100%, 2021= 97%). In Mexico, the prevalent use of gloves (2020= 98%, 2021= 99%), safety goggles (2020= 92%, 2021= 93%), disposable gowns (2020= 90%, 2021= 98%), face shields (2020= 95%), and personal protective equipment (2021= 99%) was observed.

**Table 3:** Personal protection barriers in clinical practice during COVID-19 in El Salvador and Mexico in 2020 and 2021. Statistical test: Chi-square

Groups	El Salvador 2020 (n=369) N (%)	El Salvador 2021 (n=261) N (%)	Total	P-value	Mexico 2020 (n=340) N (%)	Mexico 2021 (n=384) N (%)	Total	P-value
N95 face mask								
Yes	351 (95)	254 (97)	605	0.1544	236 (70)	289 (75)	525	0.0787
No	18 (5)	7 (3)	25		104 (30)	95 (25)	199	
Other types of face masks								
Yes	255 (69)	261 (100)	516	0.0001	143 (42)	256 (67)	399	0.0001
No	114 (31)	0	114		197 (58)	128 (33)	325	
Gloves								
Yes	369 (100)	256 (98)	625	0.0229	333 (98)	380 (99)	713	0.2629
No	0	5 (2)	5		7 (2)	4 (1)	11	
Googles or glasses								
Yes	369 (100)	250 (96)	619	0.0001	310 (92)	356 (93)	666	0.4491
No	0	11 (4)	11		30 (8)	28 (7)	58	
Disposable gown								
Yes	168 (45)	247 (95)	415	0.0001	307 (90)	373 (98)	680	0.0001
No	201 (55)	14 (5)	215		33 (10)	11 (2)	44	
Disposable surgical cap								
Yes	369 (100)	254 (97)	623	0.0004	281 (83)	342 (89)	623	0.0129
No	0	7 (3)	7		59 (17)	42 (11)	101	
Face shield								
Yes	369 (100)	253 (97)	622	0.0003	324 (95)	341 (89)	665	0.0001
No	0	8 (3)	8		16 (5)	43 (11)	59	
General Personal Protective Equipment								
Yes	86 (23)	230 (88)	316	0.0001	227 (67)	379 (99)	606	0.0055
No	283 (77)	31 (12)	314		113 (33)	5 (1)	118	

The association between dental care service, preventive measures, and protective barriers for COVID-19 in El Salvador and Mexico during 2020 and 2021 can be observed in Table 4. The use of phone triage (OR = 1.3, 95% CI = 1.05-1.66, p = 0.0163), thermometers (OR = 1.4, 95% CI = 1.09-1.82, p = 0.0074), physical distancing (OR = 1.7, 95% CI = 1.28-2.25, p = 0.0002), and face shields (OR = 2.6, 95% CI = 2.00-4.00, p = 0.0001) were significantly associated with dental care service in both countries during the COVID-19 pandemic.

**Table 4:** Binary logistic regressions for association the dental care service with the preventive measures and protection barriers during COVID-19 in El Salvador and Mexico in 2020 and 2021. Dependent variable: dental care service. Physical distancing: Space of at least one meter between patients. OR: Odds ratios. CI: Confidence intervals.

Groups	El Salvador 2020-2021 (n=630) OR	95% CI	P-value	México 2020-2021 (n=724) OR	95% CI	P-value	El Salvador México 2020-2021 (n= 1,354) OR	95% CI	P-value
Phone triage	1.7	1.17-2.50	0.0049	5	3.40-8.20	0.0001	1.3	1.05-1.66	0.0163
Infrared thermometer	-----	-----	-----	3	2.30-5.01	0.0001	1.4	1.09-1.82	0.0074
Physical distancing	1.5	1.01-2.01	0.0437	4	3.00-7.00	0.0001	1.7	1.28-2.25	0.0002
Handwashing	2	1.29-3.00	0.0019	-----	-----	-----	-----	-----	-----
Use of protection barriers									
										Disposable gown	2	1.50-3.00	0.0003	3	0.11-0.60	0.0011	-----	-----	-----
Face shield	-----	-----	-----	3	0.13-0.54	0.0001	2.6	2.00-4.00	0.0001

## Discussion

The onset of the COVID-19 pandemic necessitated modifications in clinical care and practice across the healthcare sector. This study aimed to identify the association between dental care services and the preventive measures and protective barriers employed in clinical practice in El Salvador and Mexico during the years 2020 and 2021. Given the findings, we believe it is essential to identify and analyze dental care services, preventive measures, and protective barriers in clinical settings to enhance patient care.

Health care service

During the first months of the pandemic, healthcare workers around the world seemed to have a positive attitude, good knowledge, and good management of preventive measures when facing the COVID-19 pandemic. For example, in the study by Shi et al., the authors found that in a Chinese population made up of psychiatric doctors and nurses, 90% of them had extensive knowledge about COVID-19. In addition, 60% of those surveyed had received training in the management of suspicious patients or patients with COVID-19. Likewise, almost 80% of those surveyed were willing to care for patients with COVID-19 [14].

In the American continent, health professionals also had to make changes to care services during the pandemic [15]. To minimize physical contact between hospital staff and patients, some hospitals reduced their staff presence by up to 80%. Non-urgent patient appointments were also postponed or canceled. While specialized patient care was feasible in the developed nations of the Americas, the situation was more challenging in less developed countries. Initially, healthcare workers maintained an optimistic outlook [16,17].

However, although specialized patient care could be carried out in developed countries of the American continent, the situation was more complicated in less developed countries [18]. In addition, even though at first health workers had an optimistic outlook, as the pandemic period lengthened, the positive attitude was lost and a great deal of stress and concern began to exist among health workers [19].

In the dental community, Posse et al. conducted a survey spanning 59 countries with approximately 400 participating dentists. The study reported that 80% of the dentists limited their clinical activities, with 50% attending only to dental emergencies [16]. Moraes et al. reported a cross-sectional study with a sample of Brazilian dentists. The study found that 36% of dentists suspended dental care, while 58% treated only emergencies [20]. Our survey's initial results in El Salvador and Mexico align with these findings. A significant number of dentists limited their clinical practice during the pandemic's first wave. Those who continued to provide dental care primarily treated emergencies, adhering to recommended preventive measures [16,20,21].

Moraes et al. conducted another study during the pandemic's second wave in Brazil in May 2021. This survey, which involved around 1,900 dentists, analyzed their clinical practice, knowledge, and attitudes regarding the pandemic. A third of the respondents expressed confidence in vaccine efficacy, with 96% having received at least one vaccine dose. Additionally, 27% reported having contracted COVID-19. The impact of the pandemic on their clinical practice during the second wave was less pronounced than during the first. Most felt well-prepared to treat COVID-19 patients, a sentiment that aligns with our findings in Mexico, where the majority of dentists continued treating patients while adhering to recommended preventive measures [22].

After conducting a bibliographic search, we did not find any articles related to the risk of COVID-19 infection among dentists in Mexico or El Salvador. However, in the United States, the risk of a dentist becoming infected with COVID-19, when using preventive measures, was calculated to be 1 in 13,000 [23]. Brazil was the first country in Latin America to report a positive case of COVID-19. This case was specifically in the state of São Paulo, where an approximately 60-year-old individual, who had traveled from Italy, was diagnosed with the disease [24].

Various authors in Europe and America have identified a significant number of dentists infected with COVID-19 [16]. Moraes et al. reported that, in a sample of 2,127 dentists from 11 Latin American countries, nearly 5% were found to be infected by COVID-19 [9]. In light of these findings, several researchers have published studies related to the use of preventive measures and protective barriers [25].

Preventive measures and protective barriers

Nepal et al. surveyed approximately 350 health professionals, including medical assistants, nurses, and doctors. Generally, the respondents demonstrated an intermediate to high level of knowledge about COVID-19 and exhibited an intermediate to moderate understanding of practices and prevention of COVID-19 infections [26].

In studies focusing on dentists, varied results have been reported concerning their knowledge of COVID-19 and the implementation of preventive measures. Kamate et al. conducted a survey spanning five continents to explore changes in dental practices during the COVID-19 crisis. An overwhelming 99% of the participants indicated that they adopted preventive measures against COVID-19, though the specific measures were not detailed [27]. Gambhir et al. carried out a survey involving approximately 200 dentists. While a majority displayed a comprehensive understanding of COVID-19 and its transmission routes, only 30% reported a thorough knowledge and utilization of personal protective equipment [28].

Several authors have highlighted the widespread use of preventive measures such as phone triage, infrared thermometers, handwashing, physical distancing (maintaining at least one meter between patients), and techniques to minimize aerosol generation [24,26,29]. Gold et al. noted that in US hospitals, every admitted patient underwent evaluation both over the phone and in person to ascertain potential COVID-19 infection. Those suspected were isolated for 14 days, with confirmed cases being closely monitored throughout their isolation period. Additionally, hospitals introduced measures to reduce physical interactions between doctors and patients [17]. Abed et al. executed a national web-based cross-sectional survey from November 2020 to January 2021. Their findings revealed that in Qatar, only 50% of health professionals, a majority of whom were dentists, utilized personal protective equipment. The most commonly adopted infection control measure was handwashing [30].

Balkaran et al. conducted a study across Caribbean countries using a questionnaire distributed via email. Although this study spanned several countries, it only involved 152 dentist participants. From the results, a majority of the dentists identified aerosol production during dental consultations as the primary source of contagion. Nearly 70% of the participants expressed willingness to get vaccinated, and approximately 95% reported using personal protective equipment [31].

Dentists in Mexico and El Salvador have been diligent in adhering to preventive measures and using personal protective equipment, a trend consistent with findings from previous studies [21,32].

Association between dental care service and preventive measures

The COVID-19 pandemic has significantly altered clinical care and practices. In this study, data from 1,719 dentists from El Salvador and Mexico were analyzed. The utilization of phone triage (OR = 1.3, 95% CI = 1.05-1.66, p = 0.0163), infrared thermometers (OR = 1.4, 95% CI = 1.09-1.82, p = 0.0074), physical distancing (OR = 1.7, 95% CI = 1.28-2.25, p = 0.0002), and face shields (OR = 2.6, 95% CI = 2.00-4.00, p = 0.0001) were found to be associated with dental care services during the COVID-19 period.

These findings can be utilized to a) gather information on the subject, b) reevaluate the preventive measures and protective barriers currently in use to determine their relevance in professional practice and c) develop and implement protocols aimed at addressing and mitigating challenges similar to or more complex than those posed by the COVID-19 pandemic. It is plausible that the identified variables associated with dental care services might become standard practices to enhance patient care. The current situation underscores the importance of preventive practices and minimally invasive techniques [33,34].

In the literature, there are few longitudinal studies focused on evaluating the use of COVID-19 prevention measures in dentistry and medical practice in general. However, our results align with those of other similar longitudinal studies. For instance, in June 2021, a study analyzed the prevalence and incidence of COVID-19 infection in dentists over a six-month period. The study's findings showed that dentists who maintained prevention measures, such as the use of personal protective equipment, had the lowest percentages of prevalence and incidence of COVID-19 infection over the six months evaluated [35]. Another study published in 2022 assessed the use of biosecurity measures during dental care in preventing COVID-19 infections over a three-month period. The study's results demonstrated that maintaining prevention measures significantly reduced the rates of COVID-19 infection during dental care [36]. Therefore, the results of the two aforementioned studies, along with the findings of the present study, reinforce the theory that maintaining minimum prevention measures, even after the most severe infection waves of COVID-19 could help prevent new pandemics and avoid potential COVID-19 infection outbreaks [37], which can arise from the extreme relaxation of prevention measures [38] or new virus variants that continue to emerge [39].

Limitations of the study

The study, focusing solely on El Salvador and Mexico, may not be generalizable to other countries with distinct healthcare systems or COVID-19 dynamics. Relying on a 16-question survey introduces potential biases from self-reporting and might not encompass the full scope of the topic.

## Conclusions

During 2020 and 2021, in El Salvador and Mexico, a significant association was observed between dental care services and various preventive measures and protective barriers against COVID-19. Specifically, the use of phone triage, thermometers, physical distancing, and face shields showed a positive correlation with the provision of dental services in both countries. These findings underscore the importance of national public health agencies promoting the implementation and adherence to minimum safety measures in dental care, even though the most severe waves of COVID-19 have passed. All of this is to be previously prepared to deal with new pandemics or the possible resurgence of reinfection waves that have been observed when relaxing prevention measures due to the continuous presence of new virus variants.

## Appendices

**Table 5:** Questionnaire items

Questionnaire items	
1. Gender	Male/female
2. Age	
3. In which state of the country do you reside?	
4. What decision did you make regarding your daily consultation during the pandemic?	Suspension of dental care
	Emergency only
	Dental care with preventive measures and protection barriers
5. Did you perform telephone triage?	Yes
	No
6. Did you use the infrared thermometer?	Yes
	No
7. Did you wash your hands?	Yes
	No
8. Did you carry out physical distancing?	Yes
	No
9. Did you use techniques to prevent the generation of aerosols?	Yes
	No
10. Do you use N95 face mask?	Yes
	No
11. Do you use another type of facial mask?	Yes
	No
12. Do you use gloves?	Yes
	No
13. Do you use protective glasses or Googles?	Yes
	No
14. Do you use a disposable surgical cap?	Yes
	No
15. Do you use a face shield?	Yes
	No
16. Do you use personal protective equipment?	Yes
	No
